# Association between serum estradiol level on the hCG administration day and IVF-ICSI outcome

**Published:** 2012-01

**Authors:** Mustafa Kara, Tayfun Kutlu, Kenan Sofuoglu, Belgin Devranoglu, Tansel Cetinkaya

**Affiliations:** 1 Department of Obstetrics and Gynecology, Medical Faculty, Bozok University, Yozgat, Turkey.; 2 Zeynep Kamil Women's and Children's Hospital, Reproductive Medicine and IVF Unit, Istanbul, Turkey.

**Keywords:** *In-vitro fertilization*, *Estradiol*, *Pregnancy*, *Follicle stimulating hormone*

## Abstract

**Background: **Estradiol (E_2_) is required for follicular development and play an important role in embryo implantation.

**Objective:** The aim of this study was to assess the impact of serum E_2_ levels on the day of hCG administration in IVF-ICSI patients who are performed controlled ovarian hyperstimulation (COH).

**Materials and Methods: **A total of 203 women who were undergone one time IVF cyclus were evaluated in this cross sectional study. All the patients were treated either with long protocol or with microdose flare protocol. The patients were categorized into five groups according to the serum E_2_ levels on the day of hCG administration.

**Results: **The mean number of the retrieved oocytes was (NRO) 10.6±6.7, mean fertilization rate was 55.7±24.8, and implantation rate was 9.0±19.2. Of 203 patients, 43 (21%) patients were pregnant. When the overall results are examined, the number of the retrieved oocytes and the number of transferred embryos were better in patients with serum E_2_ levels >4000 pg/ml and these values were statistically significant. There were no statistical difference in patients 37 years or older. In women ≤36 years old, the IVF-ICSI outcomes were better in patients with serum E_2 _levels >4000 pg/ml.

**Conclusion: **In spite of the lack of high quality evidence to support a positive association between serum E_2_ levels and IVF-ICSI outcomes, this study shows that high E_2_ levels during COH might be associated with an increased potential of pregnancy depending on better ovarian response. When the overall results are examined, the best scores were in patients with serum E_2_ levels >4000 pg/ml.

## Introduction

 In contemporary IVF procedure, retrieved oocyte number is positively correlated with live birth rate. As a result of this, the success which measured live birth rate is based on obtaining enough mature follicle which contains critically well oocytes (1, 2). 

Many techniques are developed to obtain a lot of follicles. During the past decade, an important increase has occurred in the use of ovulation induction regimens, mainly those using gonadotropins and GnRH analogues. COH causes to achieve multiple oocytes, but this condition results supraphysiologic E_2_ levels and might affect endometrial implantation. High E_2 _levels on the day of hCG administration might cause better IVF-ICSI outcome or a decreased outcome, caused by disrupted endometrial receptivity (3-6).

Several studies have suggested that supraphysiological E_2_ concentrations affect the probability of pregnancy. On the other hand, alternative studies indicated that high serum E_2 _concentrations do not alter endometrial receptivity (5, 7, 8). In the light of these datas, the importance of high E_2_ levels on the day of hCG administration remains controversial, in terms of IVF-ICSI outcome. We aimed to assess the association between E_2_ levels on the day of hCG administration and IVF-ICSI outcome, in which GnRH analogues were used for down regulation. 

## Materials and methods

The patients were divided into five groups according to the serum E_2_ levels at the day of hCG administration: group I (<1000 pg/ml), group II (1000-2000 pg/ml), group III (2000-3000 pg/ml), group IV (3000-4000 pg/ml) and group V (>4000 pg/ml). We retrospectively analyzed data of patients who underwent completed IVF-ICSI cycles and had a fresh embryo transfer. 

The data of the 203 patients were collected from the registrations of January 2006 to January 2008. The study included patients who received COH and had E_2 _levels on the day of hCG administration. E_2 _levels were assayed at the same labaratory. Serum E_2_ levels were assessed by enzym immuno assay and this method was designed by Abbott Laboratories, Abbott Park. The lower limit of detection for E_2_ was 53 pmol/lt. E_2_ was measured in the morning blood on the day of hCG administration. 

In all cases, pituitary was down-regulated with Leuprolide acetate (Lucrin ® daily 0.25 mg, Abbott, USA) in a long protocol or microdose flare protocol, according to patient characteristics or response during previous cycles. When the long protocol was performed Leuprolide acetate was started with 0.5 mg dose on the 21^th^ day of the previous cycle and when the pituitary supression became the dose was reduced to 0.25 mg and was continued until the day of the hCG. 

On the other hand, the same drug was started with 0.1 mg dose on the 2^nd^ day and was continued until the day of the hCG for microdose protocol. Ovulation induction was performed with HP-u FSH (Fostimon HP® 75 İBSA, Switzerland) starting on cycle day 3. Average FSH starting dose was 225 IU in the long protocol and the dose was individually adjusted according to the previous treatment cycles, body mass index (BMI), and age. When microdose protocol was used the average FSH dose was 450 IU.

Follicular development was monitorized and dose adjusment was done according to E_2 _level and ultrasonographic measurements. When at least three follicles reached 17 mm size, hCG (Pregnyl® 5000 IU×2, Schering-Plough, USA) was administered for final maturation. Transvaginal ultrasound guided needle aspiration of follicular fluid was carried out 35 to 36 hours after hCG administration. Immediately after follicle puncture, the oocytes were incubated for 2-4 hours in the incubators, and then hyaluranidase (Vitrolife, Sweden AB, Kungsbacka, Sweden) was applied for denudation procedure. 

In all cases, ICSI was performed. Semen samples were washed by using gradient method. Isolate Sperm seperation medium (Irvine Scientific, Santa Ana, California) and Quinn’s Sperm washing medium (Sage, Trumbull, CT, USA) were used for sperm preparation. G-MOPS plus, G-IVFplus, G1-plus, G2-plus (Vitrolife, Sweden AB, Kungsbacka, Sweden) were the mediums which were used for embryo culturing. Embryos were classified according to the number of blastomeres, percentage of fragmentation and blastomere appearences as type I, II, III or IV on 1st, 3rd and 5th days. 

Up to four embryos were transferred into the uterine cavity on day 2, 3 or 5 after oocyte retrieval. All transfers were made by using Rocket Thin wall Transfer set (Rocket Medical, Hingham, MA,USA). Luteal phase support was given by transvaginal progesterone administration (Crinone %8 vaginal gel ® Merck-Serono, Switzerland). Progesterone administration was initiated on the oocyte pick-up day and continued for 12 days (until the serum beta hCG measurement day). In cases of pregnancy, progesteron was given until the 12^th^. gestational week. The cycle cancellation rate was not calculated, because such patients were not included to the study. OHSS was not developed.

**Statistical analysis**

Student’s t-test, one-way analysis of variance (ANOVA) and χ^2^ tests were performed. Differences were considered significant at p<0.05. SPSS 9.05 (SPSS Inc., Chicago, IL, USA) was used for statistical analysis. The mean and standard deviation were calculated for continuous variables.

## Results

Table I shows total IVF-ICSI outcomes of the patients. The number of retrieved oocytes, number of transferred embryos, fertilization rates, implantation rates, pregnancy rates and delivey rates were assessed totally. The mean age was 30.7±4.9 years. In 43 patients (21%) clinical pregnancy were achieved but, delivering live baby rate was 15%. The number of retrieved oocytes were 10.6±6.7, the number of transferred embryos were 2.4±0.8. The fertilization and implantation rates were 55.7% and 9.0%, respectively.

**Figure 1:**
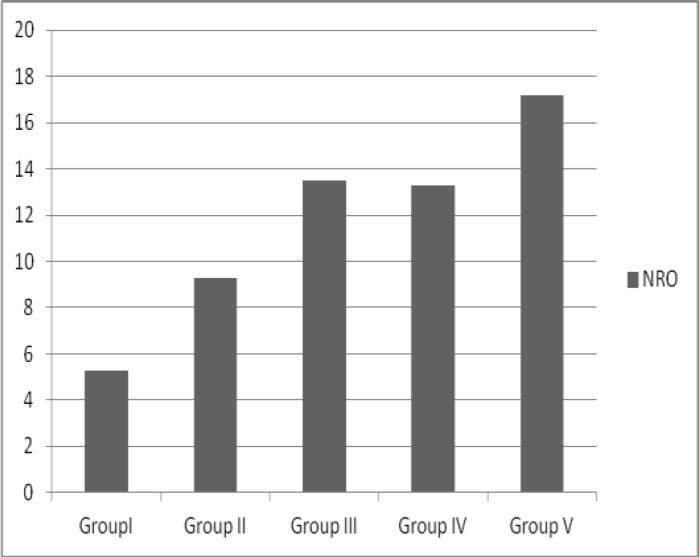
The association between NRO and serum E_2_ levels. NRO: Number of retrieved oocytes

Table II shows IVF-ICSI outcomes of the patients according to the serum E_2_ levels on the day of hCG administration. The number of retrieved oocytes and number of transferred embryos (NTE) were 17.2±4.4 and 2.5±0.6, respectively in patients with serum E_2_ levels >4000 pg/ml and these values were statistically significant (Figure 1, p<0.005). It is interesting that there were no statistically significant differences in other IVF-ICSI outcomes. The pregnancy, implantation and delivery rates were high in group V patients but, the difference between groups was not statistically significant.

**Figure 2:**
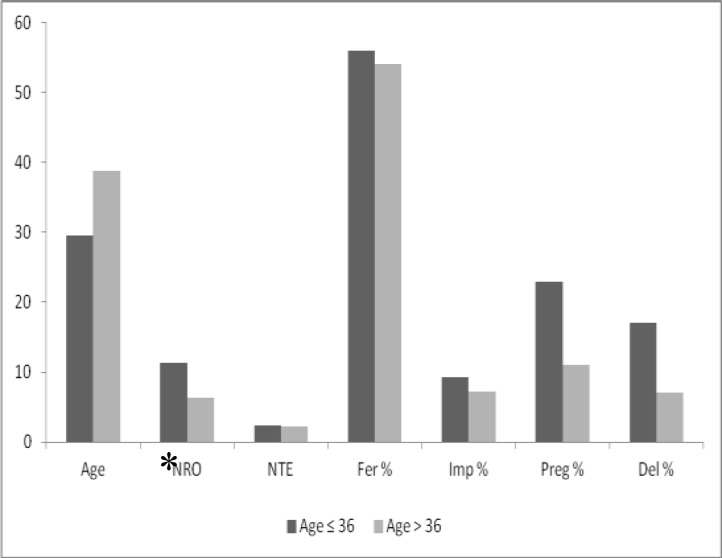
Comparison of the IVF-ICSI outcomes according to the E_2_ levels on the hCG day in patients≤ 36 years and > 36 years. NRO: Number of retrieved oocytes, NTE: Number of transferred embryos, Fer: Fertilization, Imp: Implantation, Preg: Pregnancy, Del: Delivery.^*^ means statistically significant.

We also analyzed the IVF-ICSI outcomes in 2 group according to the age of the patients: In women ≤36 years and in women >36 years. Age is one of the most important prognostic determinant and for this reason we compared potential effect of serum E_2_ level on IVF outcomes according to the age (Figure 2). As a matter of fact, the fertilization, implantation and pregnancy rates decrease in older ages. In >36 years patients IVF outcomes were lower than ≤36 years women. All parameters were better in ≤36 years patients but only number of retrieved oocyte value was statistically significant (11.7±7.3 Vs. 7.3±4.8, p<0.005).

**Table I:** Overall IVF-ICSI outcomes of the patients

**Parameters**	**IVF-ICSI Outcome**
Patient number (n)	203
Age (year)	30.7 ±4.9
Number of retrieved oocytes (NRO)	10.6 ±6.7
Number of transferred embryos (NTE)	2.4 ±0.8
Fertilization rate (%)	55.7 ±24.8
Implantation rate (%)	9.0 ±19.2
Pregnancy rate (%)	21 ±41
Delivery rate (%)	15 ±36

**Table II.** IVF-ICSI outcomes of the patients according to the serum E_2_ levels on the day of hCG administration.

** Patient number (n) **	**Group I**	**Group II**	**Group III**	**Group IV**	**Group V**
**Parameters**	**(29)**	**(88)**	**(42)**	**(33)**	**(11)**
Age (year)	31.7 ± 4.7	31.3 ± 5.1	29.9 ± 4.7	30.6 ± 4.4	27.5 ± 4.5
Number of retrieved oocytes (NRO)*	5.3 ± 3.4	9.3 ± 5.5	13.5 ± 7.1	13.3 ± 7.8	17.2 ± 4.4
Number of transferred embryos (NTE)*	1.7 ± 1.1	2.5 ± 0.7	2.5 ± 0.9	2.5 ± 0.9	2.5 ± 0.6
Fertilization rate (%)	56.5 ± 33.7	59.3 ± 24.4	51.3 ± 24.1	52.8 ± 19.8	51.5 ± 15.5
Implantation rate (%)	9.8 ± 27.5	9.6 ± 19.3	5.5 ± 12.5	8.0 ± 16.1	18.1 ± 21.6
Pregnancy rate (%)	14 ± 35	23 ± 42.1	17 ± 37.7	21 ± 41.5	45 ± 52.2
Delivery rate (%)	10 ±31	14 ± 34.5	14 ± 35.4	18 ± 39.2	36 ± 50.5

* means statistically significant.

## Discussion

IVF-ICSI is performed widely in the world and COH is essential to achieve for this. COH would improve the chance of fertilization and allow an increased number of embryos for transfer to give acceptable success rates (9-11). 

It is clear that supraphysiologic levels of E_2_ are inevitably attained during COH owing to the development of multiple ovarian follicles, each contributing significantly to E_2_ production which can reach levels up to 10 times or more those found during spontaneous cycles. The effect of such supraphysiologic E_2 _levels on the outcome of IVF-ICSI have remained controversial (12-14).

The purpose of the current study was to evaluate the association between E_2 _levels on the day of hCG administration and pregnancy achievement in IVF cycles where gonadotrophin down-regulation was used.

No data from prospective studies are available at present regarding the role of E_2_ levels on the day of hCG administration for the achievement of pregnancy. Although supraphysiologic E_2 _levels during ovarian stimulation for IVF represent one of the major deviations undergone by the female endocrine environment compared with the natural cycle, their significance for pregnancy achievement in IVF has only been assessed retrospectively (15- 17). 

Joo BS *et al* (2010) reported that there is an optimum range of serum E_2_ levels that positively affect IVF outcome. In women ≥38 years old, elevated serum E_2_ levels were more detrimental to implantation compared with in younger women. Their results suggest 3000–4000 pg/mL for women< 38 years and 2000–3000 pg/mL for women≥ 38 years as an optimal range of E_2_ levels. The present study shows that the pregnancy and implantation rates increased gradually as serum E_2_ levels increased and it was not observed a negative effect of supraphysiologic serum E_2_ level on IVF-ICSI outcome.

The association between serum E_2 _level on the hCG administration day and IVF-ICSI outcome was evaluated in many studies. Some studies do not support an association between E_2_ levels on the day of hCG administration and pregnancy achievement (8, 18, 19). The majority of studies suggested with the higher E_2_ levels on the day of hCG administration, the higher pregnancy rates achieved (1, 3- 5). 

On the contrary, three studies suggested a detrimental role of high E_2_ levels on the day of hCG administration for pregnancy achievement (2, 7, 8). Estrogen increases endometrial proliferation and uterine perfusion and because of this characteristic, estrogen improves the possibility of pregnancy. E_2_ may cause endometrial damage and disrupt the implantation and this property may be responsible for the negative effect of E_2 _on IVF-ICSI outcome. The retrospective nature of the studies might partially explain the controversy surrounding the results reported. However, this might also be due to additional confounding factors such as the small number of patients included in some studies or patients contributed more than one cycle for analysis. The restricted number of patients in our study may be criticised. But one should realise that this study reflects the results of a patients group whose members are inhabitant of low socio-economic level with low financial support to complete a successfull treatment program.

Differences in the type of analogue or in the analogue protocol used and the day that embryo transfer took place, might also have affected the results observed. The study by Chen *et al* (2003) provided some evidence that a differential association between E_2_ levels on the day of hCG administration and pregnancy rate may be present, depending on the day that embryo transfer is carried out. This might require further investigation.

The aim of this study was to evaluate the association between serum E_2 _level on the hCG administration day and IVF-ICSI outcome. The effect of serum E_2_ level on the day of hCG administration on the number of retrieved oocytes and the pregnancy rate depends on women’s age. IVF-ICSI outcomes were better in women ≤36 years old. Current state indicates that, age is important to determine success of IVF-ICSI. 

Consequently, the success of IVF-ICSI is affected by serum E_2_ level and age. When the serum E_2 _concentration reaches to supraphysiologic level, the achievement of treatment increases. But this achievement is age dependant. On the other hand, arising Estrogen more than 5000 pg/ml entertain OHSS risk. 

Therefore, taking the pregnancy rate and ovarian hyperstimulation syndrome into consideration, COH for IVF-ET should aim at an optimum rather than maximum number of oocytes without compromising uterine receptivity or embryo implantation. Our study is limited because of retrospective and small number of groups. Large and prospective randomised controlled studies are required to confirm our study.
